# Knowledge and Practice of Breast Cancer Screening Methods among Female Community Pharmacists in Jordan: A Cross-Sectional Study

**DOI:** 10.1155/2021/9292768

**Published:** 2021-09-30

**Authors:** Nehad M. Ayoub, Ghaith M. Al-Taani, Basima A. Almomani, Linda Tahaineh, Khawla Nuseir, Areej Othman, Kofi Boamah Mensah

**Affiliations:** ^1^Department of Clinical Pharmacy, Faculty of Pharmacy, Jordan University of Science and Technology (JUST), Irbid 22110, Jordan; ^2^Department of Clinical Pharmacy and Pharmacy Practice, Faculty of Pharmacy, Yarmouk University, Irbid, Jordan; ^3^Maternal and Child Health Nursing Department, School of Nursing, University of Jordan, Jordan; ^4^Department of Pharmacy Practice, Faculty of Pharmacy & Pharmaceutical Sciences, Kwame Nkrumah University of Science & Technology, Ghana

## Abstract

**Objectives:**

Our study is aimed at exploring the knowledge and personal practice of breast cancer screening among female community pharmacists in Jordan.

**Methods:**

A cross-sectional survey was carried out using a nonrandom sample selection method for pharmacists in community pharmacies.

**Results:**

A total of 551 female pharmacists completed the questionnaire. The mean age of pharmacists was 29.1 ± 7.3 years (range 21–67), and most have bachelor degrees in pharmacy (89.1%). The mean score of knowledge of breast cancer signs and symptoms was 4.2 ± 1.5 out of 6 points (range 0–6). The mean score of knowledge of risk factors was 7.6 ± 1.9 out of 12 points (ranging from 2–12). The mean score for knowledge of screening guidelines was 2.8 ± 0.9 out of 4 points (range 0–4). Overall, 452 pharmacists (85.8%) had acceptable knowledge while 75 pharmacists (14.2%) had poor knowledge of breast cancer. Pharmacists surveyed were aware of the different screening methods of breast cancer. The percentage of pharmacists who has performed breast self-examination (BSE), clinical breast examination (CBE), and mammography was 46.6%, 16.5%, and 5.4%, respectively. The most common reason for the lack of BSE and CBE performance was the absence of breast symptoms. Not being at the age recommended for mammography was the most common reason for not undergoing this screening method. Knowledge and practice of screening methods were influenced by age, years of experience, geographic region, personal history of breast cancer, and educational level among community pharmacists.

**Conclusions:**

This study revealed some gaps in the knowledge of breast cancer among female community pharmacists. The practice of the different screening methods was suboptimal, and variable reasons were indicated for the low uptake of these screening methods. Community pharmacists need to practice preventive behaviors to a satisfactory level to encourage women in the community to adopt similar behavior.

## 1. Introduction

Breast cancer is the top cancer in women in both the developed and the developing world [1]. It accounts for 30% of all newly diagnosed cancer cases among women worldwide [1]. Data from Jordan National Cancer Registry show breast cancer as the leading cancer among women accounting for 39.4% of all newly diagnosed cases [2]. Most breast cancer patients in Jordan (30.5%) are presented with advanced stages and more aggressive tumors [3]. Therefore, early detection is critical to improving breast cancer outcomes and survival.

Early diagnosis of breast cancer is a potentially beneficial way to control the disease and reduce mortality [4]. Breast self-examination (BSE), clinical breast examination (CBE), and mammography are essential for the early diagnosis of breast cancer [4]. According to the American Cancer Society (ACS) updates for breast cancer screening, mammography is currently the standard tool for early detection of disease among average-risk women [5]. Although the recent ACS guidelines no longer recommend BSE or CBE, it is noteworthy to mention that BSE allows women to recognize their breasts and identify changes if any, especially in areas where access to CBE and mammograms is challenging [6].

Healthcare workers are a direct source of medical information for the public and are essential contributors to promoting breast cancer awareness among their communities [7, 8]. Community pharmacists are readily accessible healthcare professionals who can raise awareness of breast cancer and its screening among women in the community [9]. The scope of the pharmacy profession had recently progressed towards a patient-focused approach rather than the conventional product-focused approach, enabling pharmacists to expand the range of health services provided to patients [10]. Female healthcare professionals have a significant influence on the perspective of female patients regarding screening practices, and they represent a source of positive motivation for women to enhance their awareness and uptake of screening methods for the early detection of the disease [11, 12]. Therefore, the level of knowledge, attitude, and practice of breast cancer early detection methods by healthcare professionals are key determinants of their influence on adopting screening methods by women in their communities [13, 14].

A limited number of studies have been conducted to evaluate the knowledge and practice of breast cancer screening methods among healthcare professionals in Jordan. Therefore, this study was conducted to assess the knowledge and screening practices of breast cancer among female community pharmacists in Jordan. The study is also aimed at identifying motivators for the screening behavior for the early detection of breast cancer.

## 2. Methods

### 2.1. Study Design and Population

A descriptive cross-sectional design was carried out among female pharmacists in community settings over the different directorates in Jordan. Pharmacists who have a bachelor's degree in pharmacy (BPharm) or a higher educational degree were eligible to participate in this study. The study was approved by the Institutional Review Board committee of Jordan University of Science and Technology (JUST) (research number 20180026).

### 2.2. Sampling Procedure

Convenience sampling was used to recruit community pharmacists. Pharmacists were approached by a trained research assistant who explained the purpose and procedures of the study. Pharmacists who agreed to participate in the study were handed out the questionnaire and requested to fill it in while the research assistant was available. This approach allows for consistency in answering any raised issues during data collection and improves response rate. The average time to complete the questionnaire was 5-10 minutes.

### 2.3. Data Collection Form and Scoring

A structured, self-administered questionnaire was used to collect responses. The questionnaire was developed and modified by the researchers based on previous literature and was administered to participants in the English language [12–17]. The questionnaire face and content validity were evaluated by different Faculty members at the Faculty of Pharmacy at JUST. Relevance and clarity of the survey questions were further evaluated through a pilot study (*n* = 25). Feedback and comments by the pilot group resulted in minor edits to the study tool, which was considered to improve the clarity of the survey items. Data from the pilot sample was excluded from the final analysis. The study tool was composed of three parts: (1) demographic and practice characteristics, (2) knowledge of breast cancer, and (3) practice of breast screening methods. The internal consistency reliability coefficients (Cronbach's *α*) calculated for knowledge and screening items were 0.711 and 0.853, respectively.

Demographics included items such as age, marital status, level of education, personal and/or family history of breast cancer, and years of practice. The second part of the questionnaire assessed knowledge of breast cancer signs and symptoms, risk factors, and screening guidelines with answer options of “Correct,” “Incorrect,” and “I do not know.” Breast cancer signs and symptoms, as well as risk factors, were reported according to the ACS (https://www.cancer.org). Each “Correct” response was scored 1 point, and each “Incorrect” and “I do not know” responses were both scored zero points. Six signs and symptoms were included in this part. Pharmacists who scored 0–3 points were considered to have poor knowledge of breast cancer signs and symptoms, while those with 4–6 points were determined to have an acceptable level of knowledge. Twelve items were listed on knowledge of risk factors of breast cancer. Participants who correctly recognized 7 out of the 12 stated risk factors were determined to have acceptable knowledge of risk factors while participants who identified 6 or fewer risk factors would have poor knowledge. Four items were listed on breast cancer screening representing the ACS 2017 recommendations [5]. Participants who correctly responded to three statements were determined to have acceptable knowledge of screening guidelines. An overall score for the knowledge of breast cancer was determined by combining the scores from the three parts. Thus, the maximum score that could be reported on breast cancer knowledge is 22. Pharmacists who scored 11 points or less on the overall score were classified as having poor breast cancer knowledge. The third part of the questionnaire assessed the practices of early detection of breast cancer and uptake of the different screening tools by participants. This part includes questions about BSE, CBE, and mammography along with the motivators and barriers for taking these screening tools.

### 2.4. Data Analysis

Data analysis was performed using IBM SPSS statistical package (IBM Corp. Version 23.0. Armonk, NY, USA). Descriptive statistics were used to report study variables. Continuous variables are presented as mean ± standard deviation, and categorical variables are presented as frequency and percentages (*n*, %). Pearson's Chi-square test of independence was applied to assess associations between categorical variables. Differences between groups were determined by independent student *t*-test for two-group comparisons or one-way analysis of variance (ANOVA) followed by Tukey HSD post hoc test for multiple group comparisons. Bivariate correlation analysis was performed to test for correlations between continuous variables. All *p* values were two-sided, and differences were statistically significant at *p* < 0.05.

## 3. Results

The study was conducted over ten months, from July 2018 to April 2019. Of 600 questionnaires distributed to Jordanian pharmacists, 551 questionnaires were completed yielding a response rate of 91.8%.

### 3.1. Demographics and Practice of Study Population

The mean age of community pharmacists was 29.1 ± 7.3 years (range 21–67). Most participants were single (56.7%) and had a BPharm (89.1%) (Table 1). More than half of participants graduated from public schools (64.3%) and work as staff pharmacists (57.7%) in retail community pharmacies (69.5%) (Table 1). The average years of pharmacy practice were 5.2 ± 6.1 years (ranging from 0.08–44), and the mean number of working hours per shift was 7.7 ± 1.1 (range 2–16). Almost half of the pharmacists surveyed (*n* = 284, 51.6%) reported that their oncology education at the undergraduate level was inadequate. Most pharmacists (*n* = 418, 75.9%) never attended continuous educational activities related to cancer awareness in the last two years. Other demographic and practice characteristics are shown in Table 1.

### 3.2. Knowledge of Breast Cancer Signs and Symptoms, Risk Factors, and Screening Guidelines among Community Pharmacists

“Swelling of all or part of the breast” was the most recognized sign of breast cancer by pharmacists (*n* = 447, 81.6%). Less than half of the participants (46.7%) recognized that a painful mass is not a typical finding in breast cancer (Table 2). Nipple retraction was the least recognized sign by pharmacists (69.2%). The mean score of knowledge of breast cancer signs and symptoms was 4.2 ± 1.5 points (range 0–6). Overall, 401 pharmacists (73.6%) had acceptable knowledge while 144 pharmacists (26.4%) had poor knowledge of signs and symptoms.

The majority of pharmacists correctly recognized the age of a patient (78.2%), a positive family history of the disease (97.6%), and genetic causes (93.2%) as risk factors for breast cancer (Table 2). Nevertheless, risk factors on the reproductive history were less recognized. Early menstruation was correctly recognized by approximately one-third of pharmacists (33.6%) as a potential risk factor of breast cancer, while more than half of participants (53.1%) falsely indicated early menopause as a risk factor (Table 2). Among lifestyle factors, less than half of participants (*n* = 267, 49.0%) correctly indicated obesity as a risk factor for breast cancer. The mean score of knowledge of risk factors was 7.6 ± 1.9 points (ranging from 2–12). Most pharmacists (71.2%) had an acceptable level of knowledge of risk factors.

Regarding knowledge about breast cancer screening, most pharmacists (89.3%) correctly indicated that screening with mammography reduces breast cancer mortality (Table 2). Our results showed that 222 pharmacists (40.4%) agreed to the statement “Women should undergo regular screening with mammography starting at age 55 years” which was incorrect. Most pharmacists (*n* = 457, 83.1%) correctly responded to the statement that “Women ages 45 to 54 years should be screened annually with mammography.” The mean score for pharmacists' knowledge of screening guidelines was 2.8 ± 0.9 points (range 0–4). Among the participants, 159 pharmacists (28.9%) had poor knowledge, while 391 pharmacists (71.1%) had acceptable knowledge of screening guidelines.

The overall mean score for pharmacists' knowledge of breast cancer signs and symptoms, risk factors, and screening guidelines was 14.6 ± 3.01 out of a maximum score of 22 points (range 5–22). The majority of pharmacists (*n* = 452, 85.8%) had acceptable knowledge.

### 3.3. Association of Demographic and Practice Characteristics with Knowledge of Breast Cancer among Community Pharmacists

The age of pharmacists and the number of years of practice were positively and significantly correlated with the overall score of breast cancer knowledge (*p* < 0.001, Table 3), as well as scores of knowledge of breast cancer signs and symptoms (*p* = 0.001) and risk factors (*p* = 0.001). Although the number of working hours per shift was negatively correlated with all four scores of knowledge, none of these reached statistical significance (Table 3).

**Figure 1:**
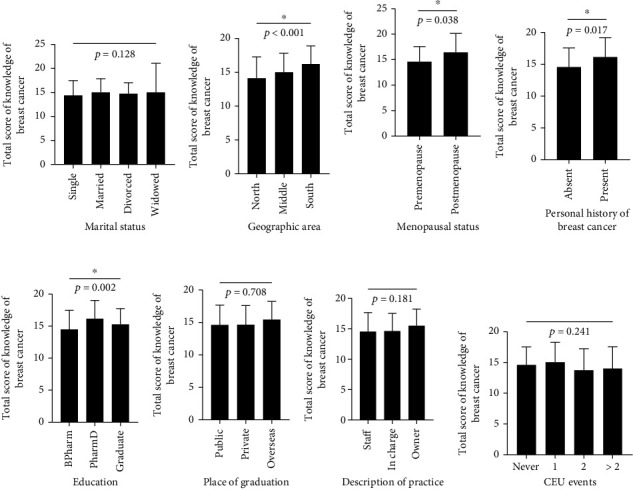
Knowledge of breast cancer based on demographic and practice characteristics of community pharmacists. Scores of breast cancer knowledge were compared according to (a) marital status, (b) geographic area, (c) menopausal status, (d) personal history of breast cancer, (e) education, (f) place of graduation, (g) description of practice, and (h) CEU events attended by community pharmacists. ∗Indicates a statistically significant difference at *p* < 0.05. BPharm: Bachelor's in Pharmacy; CEU: continuing education unit; PharmD: Doctor of Pharmacy degree.

The mean knowledge scores were significantly higher among pharmacists residing in the South compared to other geographic regions (*F* = 9.663; *p* < 0.001) (Figure 1(b)). The mean score of knowledge was significantly higher among postmenopausal compared to premenopausal participants (*t* = –2.078; *p* = 0.038) (Figure 1(c)). Similarly, community pharmacists who had a personal history of breast cancer and those with a Doctor of Pharmacy (PharmD) degree had significantly higher knowledge scores (*p* = 0.017 and *p* = 0.002, respectively, Figures 1(d) and 1(e)). Other demographic and practice characteristics lacked significant differences in knowledge level among community pharmacists in this study (Figure 1).

Associations between breast cancer knowledge with demographic and practice characteristics of pharmacists are shown in Table 4. Knowledge of breast cancer risk factors was significantly associated with the education level of community pharmacists (*p* = 0.003). In this regard, a greater proportion of pharmacists with the PharmD degree (92.9%) had an acceptable knowledge of risk factors compared to each of BPharm (68.9%) and those with graduate studies (81.3%) (Table 4). The geographic area of residence was also significantly associated with knowledge of risk factors (*p* = 0.021) and screening guidelines among community pharmacists (*p* = 0.028) (Table 4).

### 3.4. The Practice of Breast Cancer Screening Methods among Community Pharmacists

Concerning the personal practice of female pharmacists, 520 pharmacists (94.4%) were aware of BSE, and 257 (46.6%) had performed BSE. Among pharmacists who performed BSE, 68 (26.4%) performed BSE monthly, 20 (7.8%) did it twice yearly, 12 (4.7%) performed it once yearly, and 149 (57.8%) performed BSE irregularly when it comes to mind. Half of the pharmacists (*n* = 128, 50.8%) performed BSE after menstruation while a smaller number performed BSE during and before menstruation (7.1% and 8.7%, respectively). Regarding CBE, 442 pharmacists (80.5%) were aware of this screening tool. A small proportion of pharmacists had CBE performed by a healthcare professional (*n* = 73, 16.5%). Four hundred and sixty pharmacists (83.6%) were aware of mammography as a screening tool for detecting breast cancer. Sixty-one pharmacists were 40 years or older of whom 25 pharmacists had mammography performed.

**Figure 2:**
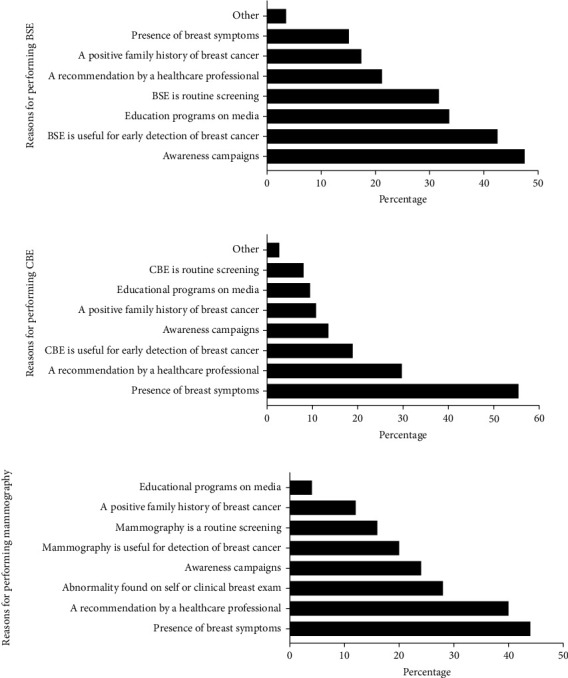
Reasons for performing the different screening methods for the early detection of breast cancer among community pharmacists.

The motivators for performing each of the screening methods are shown in Figure 2. Awareness campaigns were the most common reason (47.5%) for considering BSE (Figure 2(a)). Besides, 42.5% of pharmacists practiced BSE as they believed it is useful for the early detection of breast cancer. The presence of breast symptoms (15.1%) and a family history of the disease (17.4%) were less considered as reasons for performing BSE (Figure 2(a)). Among pharmacists who performed CBE and had mammograms, the presence of breast symptoms and healthcare professionals' recommendations was the most frequent reasons for screening (Figures 2(b) and 2(c)). Besides, 28.0% of pharmacists had mammography because of abnormal findings upon BSE or CBE. Other reasons for up-taking breast screening methods are shown in Figure 2.

**Figure 3:**
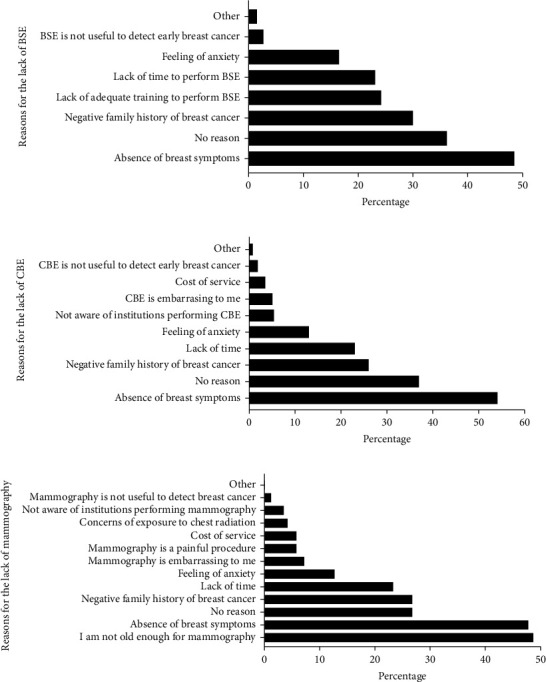
Reasons for the lack of performance for the different screening methods for the early detection of breast cancer among community pharmacists.

Among pharmacists, 263 never performed BSE. When asked the reasons, the absence of breast symptoms was most indicated (48.5%) for not considering BSE (Figure 3(a)). Notably, almost one-third of participants (36.2%) indicated no reason for not performing BSE. Additionally, 24.2% of pharmacists reported a lack of adequate training to perform BSE and 23.1% reported lack of time (Figure 3(a)). Although most pharmacists were aware of CBE, 67.0% of pharmacists (*n* = 369) had never done CBE. The most common reasons for not considering CBE included the absence of breast symptoms (54.1%), negative family history of the disease (26.0%), lack of time (23.0%), and feeling of anxiety (13.0%) (Figure 3(b)). Most pharmacists did not perform mammography, and the main reason was not reaching the appropriate age for this screening (48.7%, Figure 3(c)). Other reasons for not considering mammography were lack of time (23.3%) and that it is an embarrassing (7.2%) and painful (5.8%) procedure (Figure 3(c)).

### 3.5. Association of Screening Practices with Demographic Characteristics and Knowledge of Breast Cancer among Community Pharmacists

Screening practice was influenced by the age of the pharmacist. A greater proportion of pharmacists at age 45 or older had CBE and mammography compared to younger pharmacists (*p* = 0.001 and *p* < 0.001, respectively, Table 5). A significant association was also found between the marital status of respondents and the practice of screening methods, in which more married pharmacists tended to undergo CBE (*p* < 0.001) and mammography (*p* = 0.011) compared to single respondents (Table 5). The geographic area was also associated with the pharmacists' screening behavior. Pharmacists in middle/central region consistently practiced all screening methods to a larger extent than pharmacists in other geographic areas, and the association was statistically significant for BSE and mammography (*p* < 0.001). This study also showed a significant association between the overall level of knowledge of breast cancer and BSE practice (*p* < 0.001). The type of pharmacy, oncology education, and continuous medical education was not significantly associated with screening practice in this study (data not shown).

## 4. Discussion

Breast cancer is a global health problem that is associated with high morbidity and mortality rate in developing countries due to delayed presentation [16]. Despite the national efforts to increase awareness of breast cancer and early screening methods, population-based studies among women in Jordan showed that screening rates remain relatively low [18, 19]. Community pharmacists are in an ideal position to promote breast cancer awareness and encourage screening and early detection of the disease among women in the community [9]. To be effective educators, pharmacists themselves must possess the appropriate knowledge and screening practices for the early detection of the disease. Social and cultural factors would favor female pharmacists as the source of such information to women in the Jordanian community. In this study, we demonstrate the knowledge and practices of breast cancer screening, along with factors that might influence the screening practices among female community pharmacists.

Although the overall knowledge of breast cancer signs and symptoms was acceptable among pharmacists in this study, almost half of respondents indicated the presence of a painful mass in the breast as a sign of the disease. Nevertheless, a painless mass is a classical finding among women presenting with breast cancer, and only a few patients had varying degrees of pain or tingling [20]. Further, nipple retraction was the least recognized sign of breast malignancy among pharmacists even though abnormalities of nipple and areola are common findings in breast tumors located deep inside the nipple causing its retraction [20]. A study conducted in a university hospital in Karachi, Pakistan, revealed that most health care professionals (71%) were unaware of the fact that breast pain usually denotes a benign underlying pathology rather than breast cancer [21]. This finding was also reported among other healthcare professionals who shared the opinion that cancerous nodules in the breasts are painful [22]. In this study, the knowledge of breast cancer risk factors was acceptable among community pharmacists. However, a deficiency in recognizing the female reproductive history and lifestyle factors was noted. Modifiable risk factors such as obesity and lack of physical activity were less recognized compared to hereditary and familial factors. The lack of proper recognition for the reproductive and lifestyle risk factors was also found in studies conducted among nurses in Nigeria [16, 23]. In addition, a third of health care professionals in Pakistan was not aware of obesity as a potential risk factor [21]. According to the 2017 ACS guidelines for the early detection of breast cancer, women with an average risk of the disease are recommended to undergo regular screening with mammography starting at age 45 years [5], a statement that was not recognized by 40.4% of pharmacists in this study. The guidelines also recommend annual screening with mammography for women at age 45–55 years and every other year screening for women at 55 years of age or older [5]. Most pharmacists in this study were aware of the rate of mammography screening, and the knowledge in this regard was acceptable. Taken together, the overall knowledge of breast cancer was acceptable in most of the pharmacists surveyed (85.8%). Earlier studies of breast cancer knowledge in healthcare professionals revealed variable results. In a study by Nguefack et al. in Cameroon, less than half of the health care professionals surveyed had good knowledge of breast cancer [13]. Similarly, other studies on knowledge of breast cancer among nurses in India and Nigeria showed modest knowledge [24].

Breast self-examination (BSE) is a simple, quick, noninvasive, and inexpensive procedure for the early detection of breast cancer among women [6, 16]. BSE has the advantages of being easy to perform, convenient, and private [25]. The ACS no longer recommends monthly BSE for the early detection of breast cancer as it increases anxiety, the proportion of breast biopsies, biopsies for benign lesions, and healthcare costs [16, 21]. Nevertheless, more than 90% of the cases of breast masses are self-detected by women themselves through BSE [16]. Thus, the potential to recognize changes in the breast is better achieved when women perform BSE regularly [6]. In our study, less than half of pharmacists practice BSE, and less than a third performed the screening regularly monthly. This is much lower than the rate for BSE in other studies among female health care professionals in Cameroon [13], King Saudi Arabia (KSA) [26], Nigeria [23], Turkey [14], and Morocco [11]. However, our findings were comparable with a study conducted among female healthcare workers in a public health facility in Ethiopia showing low rates of BSE (32.6%) [25]. Similarly, the BSE practice among nurses in a teaching hospital in Nigeria was poor with only a third (31.8%) performed monthly BSE [16]. The differences in the rates of performing BSE could be due to the difference in the study period and population.

Awareness campaigns and believing in the usefulness of BSE for the early detection of breast cancer were the most reported reasons for practicing BSE among community pharmacists in this study. In other studies, however, the commonest reasons reported by female healthcare workers practicing BSE were for the early detection and the fear of developing breast cancer [25]. Reasons that may attribute to the low rates of BSE performance among women include the absence of signs and symptoms, lack of time, forgetting the BSE schedule, fear of possible discovery of a lump, embarrassment, and lack of a recommendation by a healthcare professional [4, 6, 25]. In our study, the absence of breast symptoms was the most indicated reason for not practicing BSE. However, a reasonable proportion of pharmacists (36.2%) reported no reason for the lack of BSE. This rate was similar to a study by Shallo and Boru in which 27% of the female health workers did not practice BSE because of negligence [25]. Another potential reason for the low practice of BSE among pharmacists is their knowledge of the current ACS guidelines in which BSE is no longer a recommended screening method.

Clinical breast examination (CBE) is the inspection of a female's breasts by a trained healthcare professional, such as a breast surgeon, a family physician, or a breast care nurse, to recognize different types of abnormalities in the breast [27]. Only 16.5% of pharmacists had CBE in this study. This rate was comparable to studies by Ghanem et al. (26.1%) and Heena et al. (24.1%) done in Morocco and KSA [11, 26], respectively, however, it was very low compared to the CBE level reported by Nguefack et al. in Cameroon (49.2%) [13]. Most pharmacists who had CBE did so because of the presence of breast symptoms, a similar finding in another study [11].

Mammography is the only screening modality that has been proven to reduce breast cancer mortality based on multiple randomized clinical trials [28]. It is thus the gold standard for the early detection of breast cancer [28]. Most pharmacists in the present study were aware of mammography; however, only 25 pharmacists had mammograms. This finding can be explained by the younger population of respondents in our study who are still below the age to undergo mammography. Given the ACS recommendations that women over 40 years can start regular screening with mammography [5], we analyzed the practices of the participants over the age of 40. Sixty-one pharmacists were 40 years or above, and 40.9% of pharmacists in this age group had mammography at least once. The overall rate of undergoing mammography in our study (5.4%) was lower than those found by similar studies conducted in Nigeria (8%), Morocco (15%), KSA (18.7%), Turkey (25.7%), and Cameroon (43.0%) [11, 13, 23, 26, 29]. In a study by Nazzal et al., mammography screening was assessed among 299 female healthcare workers in Palestine showing that 50% of those aged 40 years and above had at least one mammogram [30].

In the present study, the motivators for considering mammography were the presence of breast symptoms and a recommendation by a healthcare provider. Alternatively, the perceived benefit that mammography enables early detection of breast cancer was the main motivator to perform mammography by female healthcare workers in another study [30]. Several barriers were identified for not undergoing mammograms such as the pain and embarrassment associated with the procedure, fear of results, lack of health insurance, low income, poor knowledge about breast cancer screening, lack of perceived need, and lack of physician recommendation [31, 32]. In this study, most pharmacists did not have mammography because they were not at the age for this screening. This reason was also observed among other healthcare workers for not undergoing mammography in different studies [26, 29]. In the study by Nazzal et al., being busy was the most common barrier for the performance of mammography among primary female healthcare workers [30]. In our study, 26.8% of pharmacists provided no reason for not performing mammography, a finding that was similar to female healthcare workers in KSA in which 24.0% did not believe there was a reason to have mammography [26].

In this study, breast cancer knowledge was influenced by age and years of experience among community pharmacists. The level of knowledge was improved among pharmacists in South regions, postmenopausal, and those with PharmD degrees. Screening practice was influenced by age, marital status, the geographic area for respondents, and the presence of a family or personal history of the disease. Older pharmacists had better knowledge and practice screening methods to a greater extent than younger ones. Pharmacists who have a personal or family history of breast cancer also practiced these methods more than others. In a study by Soylar et al., age and family history of any cancer type were significantly correlated to the practice of cancer screening methods [29]. Knowledge of breast cancer influenced the practice of BSE in this group of community pharmacists in which pharmacists who practiced BSE had better knowledge of breast cancer. Knowledge of breast cancer stems from self-perception of the disease, which influences BSE behaviors. The pharmacists' knowledge may be due to increased awareness of the disease due to introspection. This finding agreed with other studies that showed a significant association between the level of knowledge about breast cancer and BSE practice [17, 23, 25]. Alternatively, Nguefack et al. reported a lack of impact of knowledge on screening behavior of breast cancer [13].

Most studies published on breast cancer knowledge and the screening behavior involved medical doctors, resident doctors, nurses, paramedics, midwives, and other health care professionals. Nevertheless, community pharmacists were largely underrepresented in the literature. It is worth mentioning that a previous study conducted among community pharmacists in Jordan revealed adequate knowledge along with positive attitudes and willingness of pharmacists to participate actively in promoting awareness on breast cancer [9]. Therefore, community pharmacists can educate women about breast cancer signs and risk factors, types of screening practices available, and their guidelines. Nevertheless, the low rate of screening practice observed among female pharmacists in this study might bring into question their potential as effective educators for women in the community to positively influence screening behaviors and encourage early detection of breast cancer.

This study has a few limitations. First, the self-reported information is subject to social desirability bias. Second, the questionnaire was distributed using nonrandom sampling. Convenience sampling might trigger selection bias and further limit the generalizability of the results. However, we are hopeful that the high response rate achieved in this study would mitigate the bias that may be caused because of the sampling applied.

## 5. Conclusions

To the best of our knowledge, this is the first study to assess the practice of breast cancer screening among female community pharmacists in Jordan. This study revealed the gaps in the knowledge of breast cancer among pharmacists especially regarding modifiable risk factors that can be altered to reduce the risk of breast cancer among women. Improving the knowledge of signs and symptoms and risk factors is mandatory in the curricula of undergraduate studies and through continuing medical education events and activities. While the female pharmacists in this study were aware of breast cancer screening methods and guidelines, this was not reflected in their practices. The practice of the different screening methods was inadequate, and variable reasons were indicated for the low uptake of these screening methods. Community pharmacists need to perform the preventive behaviors themselves to a satisfactory level to encourage women in the community to adopt similar behavior.

## Figures and Tables

**Table 1:** Demographic and practice characteristics of pharmacists.

Characteristic	*n* (%)
Marital status	
Single	312 (56.7)
Married	227 (41.3)
Divorced	7 (1.3)
Widowed	4 (0.7)
Geographic area	
North Jordan	269 (48.8)
Middle Jordan	254 (46.1)
South Jordan	28 (5.1)
Educational level	
BPharm	491 (89.1)
PharmD	44 (8.0)
Graduate degree	16 (2.9)
Place of graduation	
Public	353 (64.3)
Private	184 (33.6)
Overseas	11 (2.0)
Type of pharmacy	
Chain	167 (30.5)
Independent	381 (69.5)
Description of practice	
Staff pharmacist	317 (57.7)
Pharmacist in charge	193 (35.2)
Owner	39 (7.1)
Menopausal status	
Premenopausal	536 (97.6)
Postmenopausal	13 (2.4)
Smoking status	
Current	30 (5.5)
Past	9 (1.6)
Never	511 (92.9)
Personal history of breast cancer	
Yes	24 (4.4)
No	526 (95.6)
Family history of breast cancer	
Yes	78 (14.2)
No	469 (85.1)
I do not know	4 (0.7)
Personal monthly income	
<500 JD	408 (75.6)
500–1000 JD	125 (23.1)
>1000 JD	7 (1.3)
Oncology education in the undergraduate degree	
Adequate	65 (11.8)
Fair	201 (36.5)
Inadequate	284 (51.6)
Attendance of continuing education events related to cancer awareness in the last two years	
None	418 (75.9)
1 CEU	102 (18.5)
2 CEUs	24 (4.4)
>2 CEUs	7 (1.3)

BPharm: Bachelor's in Pharmacy; CEU: Continuing education unit; PharmD: Doctor of Pharmacy degree.

**Table 2:** Knowledge of signs and symptoms, risk factors, and screening guidelines of breast cancer among pharmacists.

Statement	Answer	Response *n* (%)		
		Correct	Incorrect	I do not know
Signs and symptoms				
A painful mass	Incorrect	271 (49.5)	256 (46.7)	21 (3.8)
Swelling of all or part of a breast	Correct	447 (81.6)	61 (11.1)	40 (7.3)
Skin irritation or dimpling	Correct	401 (73.3)	60 (11.0)	86 (15.7)
Breast or nipple pain	Correct	409 (74.8)	85 (15.5)	53 (9.7)
Nipple retraction	Correct	380 (69.2)	34 (6.2)	135 (24.6)
Nipple discharge	Correct	421 (76.7)	40 (7.3)	88 (16.0)
Risk factors				
Increasing age of a woman	Correct	426 (78.2)	82 (15.0)	37 (6.8)
Presence of family history of breast cancer	Correct	535 (97.6)	11 (2.0)	2 (0.4)
Genetic factors	Correct	508 (93.2)	22 (4.0)	15 (2.8)
Early menstruation	Correct	183 (33.6)	146 (26.8)	215 (39.5)
Early menopause	Incorrect	290 (53.1)	95 (17.4)	161 (29.5)
Young age at birth of first child	Incorrect	97 (17.9)	244 (45.0)	201 (37.1)
Giving birth to many children	Incorrect	81 (15.0)	301 (55.6)	159 (29.4)
Breastfeeding	Incorrect	60 (11.0)	424 (78.1)	59 (10.9)
Obesity	Correct	267 (49.0)	154 (28.3)	124 (22.8)
Alcohol intake	Correct	399 (72.9)	61 (11.2)	87 (15.9)
Lack of physical activity	Correct	294 (53.8)	112 (20.5)	140 (25.6)
Use of hormone replacement therapy	Correct	476 (87.0)	27 (4.9)	44 (8.0)
Screening guidelines				
Screening with mammography reduces mortality from breast cancer	Correct	491 (89.3)	28 (5.1)	31 (5.6)
Women should undergo regular screening with mammography starting at age 55 years	Incorrect	222 (40.4)	283 (51.5)	45 (8.2)
Women ages 45 to 54 years should be screened annually with mammography	Correct	457 (83.1)	41 (7.5)	52 (9.5)
Women aged 55 years or older should transition to screening every other year with mammography	Correct	312 (56.7)	127 (23.1)	111 (20.2)

**Table 3:** Correlations between demographic and practice characteristics and knowledge of breast cancer among community pharmacists.

Characteristics	Total score of knowledge		Score for knowledge of signs and symptoms		Score for knowledge of risk factors		Score for knowledge of screening guidelines	
	*r*	*p* value	*r*	*p* value	*r*	*p* value	*r*	*p* value
Age, years	0.168	<0.001^∗^	0.147	0.001^∗^	0.140	0.001^∗^	0.050	0.241
Years of practice	0.140	0.001^∗^	0.128	0.003^∗^	0.121	0.005^∗^	0.009	0.841
Working hours per shift	–0.081	0.065	–0.074	0.084	–0.066	0.133	–0.024	0.577

*r*: Pearson's correlation coefficient. ^∗^Indicates statistical significance at *p* < 0.05.

**Table 4:** Association of knowledge of breast cancer with demographic and practice characteristics among community pharmacists.

Characteristics	Knowledge of signs and symptoms			Knowledge of risk factors			Knowledge of screening guidelines		
	Poor (*n* = 144)	Acceptable (*n* = 401)	*p* value	Poor (*n* = 153)	Acceptable (*n* = 378)	*p* value	Poor (*n* = 159)	Acceptable (*n* = 391)	*p* value
Marital status			0.828			0.245			0.833
Single	85 (27.6)	223 (72.4)		95 (31.8)	204 (68.2)		90 (28.8)	222 (71.2)	
Married	57 (25.3)	168 (74.7)		54 (24.3)	168 (75.7)		65 (28.8)	161 (71.2)	
Divorced	1 (14.3)	6 (85.7)		3 (42.9)	4 (57.1)		2 (28.6)	5 (71.4)	
Widowed	1 (25.0)	3 (75.0)		1 (33.3)	2 (66.7)		2 (50.0)	2 (50.0)	
Geographic area			0.079			0.021^∗^			0.028^∗^
North	82 (30.7)	185 (69.3)		84 (31.9)	179 (68.1)		91 (33.8)	178 (66.2)	
Middle	55 (22.0)	195 (78.0)		67 (27.9)	173 (72.1)		59 (23.3)	194 (76.7)	
South	7 (25.0)	21 (75.0)		2 (7.1)	26 (92.9)		9 (32.1)	19 (67.9)	
Menopausal status			0.357			0.765			0.633
Premenopausal	142 (26.8)	388 (73.2)		150 (29.0)	368 (71.0)		156 (29.2)	379 (70.8)	
Postmenopausal	2 (15.4)	11 (84.6)		3 (25.0)	9 (75.0)		3 (23.1)	10 (76.9)	
Personal history of breast cancer			0.048^∗^			0.087			0.369
Yes	2 (8.7)	21 (91.3)		3 (13.0)	20 (87.0)		5 (20.8)	19 (79.2)	
No	142 (27.3)	379 (72.7)		150 (29.6)	357 (70.4)		154 (29.3)	371 (70.7)	
Educational level			0.446			0.003^∗^			0.317
BPharm	131 (27.0)	355 (73.0)		147 (31.1)	326 (68.9)		143 (29.2)	347 (70.8)	
PharmD	8 (18.6)	35 (81.4)		3 (7.1)	39 (92.9)		14 (31.8)	30 (68.2)	
Graduate degree	5 (31.3)	11 (68.8)		3 (18.8)	13 (81.3)		2 (12.5)	14 (87.5)	
Place of graduation			0.746			0.471			0.128
Public	96 (27.5)	253 (72.5)		93 (27.2)	249 (72.8)		111 (31.4)	242 (68.6)	
Private	45 (24.6)	138 (75.4)		57 (32.2)	120 (67.8)		47 (25.7)	136 (74.3)	
Overseas	3 (30.0)	7 (70.0)		3 (33.3)	6 (66.7)		1 (9.1)	10 (90.9)	
Description of practice			0.435			0.046^∗^			0.954
Staff pharmacist	86 (27.6)	226 (72.4)		85 (28.3)	215 (71.7)		93 (29.3)	224 (70.7)	
Pharmacist in charge	50 (26.0)	142 (74.0)		63 (33.0)	128 (67.0)		54 (28.1)	138 (71.9)	
Owner	7 (17.9)	32 (82.1)		5 (13.2)	33 (86.8)		11 (28.2)	28 (71.8)	
CEU			0.981			0.121			0.336
Never	108 (26.0)	307 (74.0)		117 (29.0)	286 (71.0)		128 (30.7)	289 (69.3)	
1	27 (27.3)	72 (72.7)		23 (23.2)	76 (76.8)		23 (22.5)	79 (77.5)	
2	7 (29.2)	17 (70.8)		10 (41.7)	14 (58.3)		7 (29.2)	17 (70.8)	
>2	2 (28.6)	5 (71.4)		3 (60.0)	2 (40.0)		1 (14.3)	6 (85.7)	

Data presented as *n* (%). ^∗^Indicates statistical significance at *p* < 0.05. BPharm: Bachelor's in Pharmacy; CEU: continuing education unit; PharmD: Doctor of Pharmacy degree.

**Table 5:** Association of screening practices with demographic characteristics and knowledge of breast cancer among community pharmacists.

Characteristics	BSE			CBE			Mammography		
	Yes (*n* = 257)	No (*n* = 261)	*p* value	Yes (*n* = 73)	No (*n* = 367)	*p* value	Yes (*n* = 25)	No (*n* = 435)	*p* value
Age, years			0.180			0.001^∗^			<0.001^∗^
<45	238 (92.6)	249 (95.4)		62 (84.9)	350 (95.4)		16 (64.0)	413 (95.4)	
≥45	19 (7.4)	12 (4.6)		11 (15.1)	17 (4.6)		9 (36.0)	20 (4.6)	
Marital status			0.77			<0.001^∗^			0.011^∗^
Single	129 (50.4)	159 (60.5)		21 (29.2)	218 (59.1)		6 (25.0)	237 (54.5)	
Married	123 (48.0)	98 (37.3)		49 (68.1)	144 (39.0)		17 (70.8)	188 (43.2)	
Divorced	3 (1.2)	3 (1.1)		1 (1.4)	4 (1.1)		0 (0.0)	7 (1.6)	
Widowed	1 (0.4)	3 (1.1)		1 (1.4)	3 (0.8)		1 (4.2)	3 (0.7)	
Educational level			0.031^∗^			0.290			0.166
BPharm	230 (89.5)	234 (89.0)		65 (89.0)	330 (89.4)		25 (100.0)	380 (87.4)	
PharmD	16 (6.2)	26 (9.9)		4 (5.5)	30 (8.1)		0 (0.0)	39 (9.0)	
Graduate degree	11 (4.3)	3 (1.1)		4 (5.5)	9 (2.4)		0 (0.0)	16 (3.7)	
Geographic area			<0.001^∗^			0.128			<0.001^∗^
North	104 (40.5)	147 (55.9)		25 (34.2)	165 (44.7)		2 (8.0)	204 (46.9)	
Middle	143 (55.6)	98 (37.3)		45 (61.6)	180 (48.8)		22 (88.0)	204 (46.9)	
South	10 (3.9)	18 (6.8)		3 (4.1)	24 (6.5)		1 (4.0)	27 (6.2)	
Personal history of breast cancer			0.003^∗^			<0.001^∗^			<0.001^∗^
Yes	19 (7.4)	5 (1.9)		20 (27.4)	2 (0.5)		12 (48.0)	9 (2.1)	
No	238 (92.6)	258 (98.1)		53 (72.6)	367 (99.5)		13 (52.0)	425 (97.9)	
Family history of breast cancer			0.001^∗^			0.780			0.008^∗^
Yes	54 (21.0)	24 (9.1)		12 (16.4)	55 (14.9)		9 (36.0)	59 (13.6)	
No	201 (78.2)	237 (90.1)		61 (83.6)	312 (84.6)		16 (64.0)	372 (85.5)	
I do not know	2 (0.8)	2 (0.8)		0 (0.0)	2 (0.5)		0 (0.0)	4 (0.9)	
Knowledge			<0.001^∗^			0.191			0.276
Poor	20 (8.1)	51 (20.4)		5 (7.2)	45 (12.8)		1 (4.3)	49 (11.8)	
Acceptable	226 (91.9)	199 (79.6)		64 (92.8)	306 (87.2)		22 (95.7)	368 (88.2)	

Data presented as *n* (%). ^∗^Indicates statistical significance at *p* < 0.05.

## Data Availability

The data presented in this study can be made available upon reasonable request from the corresponding author.
